# Effects of chemical composition on the lung cell response to coal particles: Implications for coal workers' pneumoconiosis

**DOI:** 10.1111/resp.14246

**Published:** 2022-03-20

**Authors:** Yong Song, Katherine Southam, B. Basil Beamish, Graeme R. Zosky

**Affiliations:** ^1^ Menzies Institute for Medical Research College of Health and Medicine, University of Tasmania Hobart Tasmania Australia; ^2^ B3 Mining Services Pty Ltd Brisbane Queensland Australia; ^3^ Tasmanian School of Medicine, College of Health and Medicine University of Tasmania Hobart Tasmania Australia

**Keywords:** coal chemistry, coal workers' pneumoconiosis, cytotoxicity, fibroblast response, inflammatory response

## Abstract

**Background and objective:**

Coal mine dust has a complex and heterogeneous chemical composition. It has been suggested that coal particle chemistry plays a critical role in determining the pathogenesis of coal workers' pneumoconiosis (CWP). In this study, we aimed to establish the association between the detrimental cellular response and the chemical composition of coal particles.

**Methods:**

We sourced 19 real‐world coal samples. Samples were crushed prior to use to minimize the impact of particle size on the response and to ensure the particles were respirable. Key chemical components and inorganic compounds were quantified in the coal samples. The cytotoxic, inflammatory and pro‐fibrotic responses in epithelial cells, macrophages and fibroblasts were assessed following 24 h of exposure to coal particles. Principal component analysis (PCA) and stepwise regression were used to determine which chemical components of the coal particles were associated with the cell response.

**Results:**

The cytotoxic, inflammatory and pro‐fibrotic response varied considerably between coal samples. There was a high level of collinearity in the cell responses and between the chemical compounds within the coal samples. PCA identified three factors that explained 75% of the variance in the cell response. Stepwise multiple regression analysis identified K_2_O (*p* <0.001) and Fe_2_O_3_ (*p* = 0.011) as significant predictors of cytotoxicity and cytokine production, respectively.

**Conclusion:**

Our data clearly demonstrate that the detrimental cellular effects of exposure to coal mine dusts are highly dependent on particle chemistry. This has implications for understanding the pathogenesis of CWP.

## Abstract 2

To determine whether chemistry is a key determinant of the lung cell response to coal particles, we examined the correlation between the chemical constituents of 19 coal samples and cellular response. We identified K_2_O and Fe_2_O_3_ as the constituents of the coal associated with the greatest detrimental cell response.

## INTRODUCTION

Inhalation of respirable coal mine dust particles can lead to coal workers' pneumoconiosis (CWP). CWP is one of the most prevalent occupational lung diseases in industrialized countries^1^, ^2^ and international data suggest an increased identification of cases in recent years.^1^, ^2^, ^3^ For example, the prevalence of CWP in miners in the United States has consistently increased from 2000 to 2017 and exceeds 10% in workers with more than 25 years of exposure.^3^

The initiation and progression of CWP is driven by coal mine dust‐induced cytotoxicity, inflammation, oxidative stress and fibrosis.^4^, ^5^, ^6^ It is generally thought that epithelial cells and resident macrophages induce persistent inflammation and tissue injury leading to a cycle of aberrant repair which promotes fibrosis.^5^, ^7^ Disease severity is well known to be correlated with mass concentration, exposure duration and particle size,^5^, ^8^ with some evidence to suggest that submicron and nanoparticles may exhibit enhanced toxicity compared to larger particles.^9^, ^10^ However, the prevalence and severity of CWP differ geographically, despite comparable exposure to respirable dust.^11^ This observation suggests that coal composition plays a key role in determining CWP pathogenesis.

This notion is supported by epidemiological studies showing correlations between coal seam geochemistry and the prevalence of CWP^12^ and early *in vitro* studies showing that cell growth is inhibited to a greater extent by leachates of coal collected from areas with a higher incidence of CWP.^8^ Recent *in vitro* studies have focused on exploring non‐carbon components of coal mine dust as the key reactive compounds leading to coal‐induced lung injury.^7^, ^12^, ^13^, ^14^, ^15^, ^16^ However, data from these studies are contradictory, highlighting the necessity for new studies that consider all components of coal mine dusts and how these impact responses in a range of cell types.

Coal mine dust is a complex mixture containing different proportions of minerals, trace metals and organics.^11^, ^17^ Given that CWP is an untreatable lung disease,^1^ a clear understanding of which components are aetiological factors for the development of disease will enable identification of the most hazardous working environments, and potentially inform the identification of new treatment strategies.

We hypothesized that the toxic effects of coal particles on lung cells are highly dependent on their organic and inorganic constituents. We aimed to establish the association between the detrimental cellular response and chemical composition of coal particles to identify key chemical factor(s) contributing to disease development.

## METHODS

### Coal samples

Nineteen Australian coal samples were sourced and crushed. Particle size was analysed using a Hitachi SU‐70 field emission analytical scanning electron microscope and calculated using the trainable Weka Segmenter plugin in ImageJ.^18^

### Coal chemistry and composition

All of the samples were analysed for proximate analysis (moisture, ash, volatile matter and fixed carbon) as well as ultimate analysis (C, H, N, S, O) and calorific value. Coal samples ranged from high‐volatile bituminous to medium‐volatile bituminous and covered a wide spectrum of ash content (8.5%–89.4%) representing a range of samples from the roof to the floor of the seam. The inorganic constituents of the samples were determined by x‐ray fluorescence.

### Cell culture

Human alveolar epithelial cells (A549; ATCC), macrophages (THP‐1; ATCC) and fibroblasts (CRL‐1490; ATCC) were cultured in Ham's F‐12K medium (21127030; Gibco) supplemented with foetal bovine serum (FBS, 10%) and glutamine (1%), growth medium (RPMI‐1640; ATCC) with 10 μM Phorbol 12‐myristate 13‐acetate (Sigma) and 10% FBS, and Eagle's Minimum Essential Medium (ATCC) with 10% FBS, respectively. All cells were seeded at 2 × 10^5^ cells/ml prior to exposure to coal particles at concentrations of 0 or 200 μg/ml^7^ for 24 h at 37°C in humidified 5% CO_2_. This timepoint was chosen on the basis of preliminary data showing a peak in cytokine production at 24 h in the absence of excessive cell death (Figure S1 in the Supporting Information).

### Quantification of cellular response

Relative cytotoxicity was assessed by lactate dehydrogenase assay (G1780; Promega) and expressed as a value relative to control. The production of inflammatory cytokines (IL‐8, IL‐6, TNF‐α and IL‐18) by the cells was quantified in the supernatant by ELISA (R&D Systems). Cell proliferation and collagen production were assessed by WST‐1 assay (ab155902; Abcam) and Sircol Soluble Collagen Assay (S5000; Biocolor), respectively. All experiments were repeated six times (fresh cell and coal preparations on different days) to allow statistical comparisons to be made between samples.

### Statistical analysis

Differences in cellular responses were analysed using one‐way repeated measures ANOVA with a Tukey honestly significant difference post hoc test using SigmaPlot (v. 13; Systat Software, San Jose, CA). Data were log_2_‑transformed to satisfy the assumptions of homoscedasticity and normal distribution of the error terms as appropriate. Due to the collinearity between the cellular responses, principal component analysis (PCA) was conducted to characterize the overall cellular response (version 20.0; SPSS Inc., Chicago, IL, USA). Pearson correlation coefficients were calculated, and stepwise multiple regression analysis was used to evaluate the association between coal chemistry and the cellular response. Significant associations identified from stepwise multiple regression analysis were further assessed by linear regression with adjustment for particle size as a co‐variate. Data are presented as mean (SD). Differences were considered statistically significant if *p* <0.05.

## RESULTS

### Particle characterization

**FIGURE 1:**
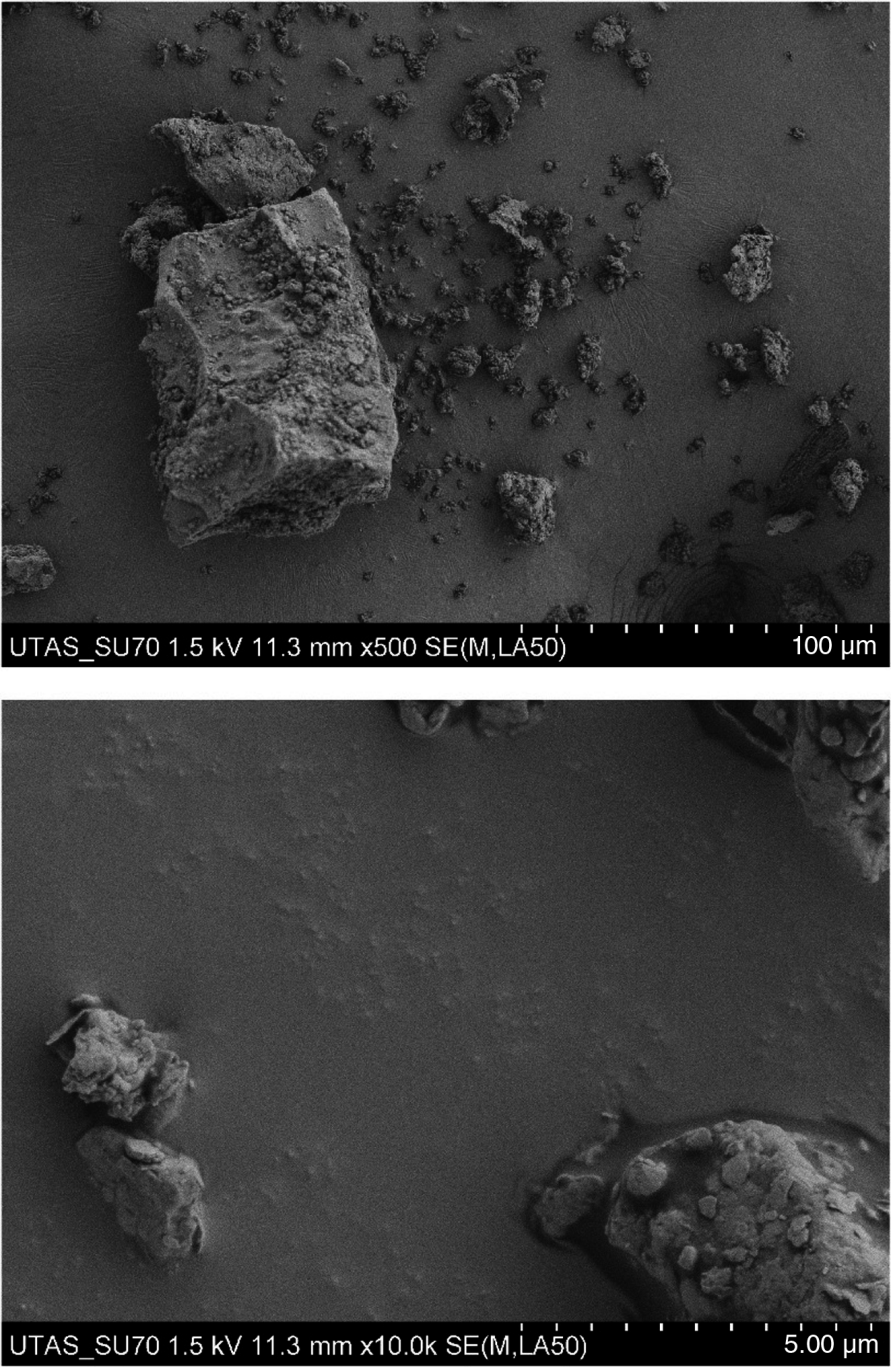
Representative images showing particle size at two different magnifications (scale bars represent 100 and 5 μm)

All coal samples were homogeneous in terms of particle size with the bulk of particles within the respirable size fraction (*p* >0.05; Table 1, Figure 1) likely a reflection of the consistent crushing process. In contrast, there were considerable variations in the chemistry of the coal samples (Table 1). The four most abundant inorganic constituents were SiO_2_ (28.77%), Al_2_O_3_ (9.40%), Fe_2_O_3_ (1.42%) and K_2_O (0.81%).

**TABLE 1:** Summary of the variation in particle size and coal chemistry between the 19 samples

	Mean	Minimum	Maximum	SD
Proximate analysis (air‐dried basis)				
Moisture (%)	5.31	2.30	10.00	2.30
Ash (%)	42.36	8.53	89.38	25.89
Volatile matter (%)	18.27	4.53	27.20	5.61
Fixed carbon (%)	34.06	0.69	64.53	21.08
Calorific value (MJ/kg, raw)	17.27	0.50	31.80	9.99
Ultimate analysis (wt%, dry ash‐free basis)				
Carbon	42.88	2.48	77.78	23.88
Hydrogen	2.77	0.93	4.51	1.04
Nitrogen	0.93	0.05	1.55	0.50
Sulphur	0.32	0.03	0.78	0.20
Oxygen	5.44	1.66	8.22	1.73
Inorganic constituents (wt%, air‐dried basis)				
SiO_2_	28.77	3.81	64.88	18.78
Al_2_O_3_	9.40	1.69	25.46	6.30
Fe_2_O_3_	1.42	0.15	4.77	1.37
CaO	0.38	0.05	0.94	0.23
MgO	0.42	0.06	1.24	0.42
Na_2_O	0.28	0.01	1.23	0.37
K_2_O	0.81	0.02	3.54	0.95
TiO_2_	0.32	0.08	0.72	0.20
Mn_3_O_4_	0.01	0.00	0.05	0.01
SO_3_	0.29	0.00	0.96	0.27
P_2_O_5_	0.05	0.00	0.27	0.08
BaO	0.10	0.00	1.38	0.32
SrO	0.01	0.00	0.04	0.01
ZnO	0.00	0.00	0.01	0.00
Particle size (µm)	0.11	0.10	0.12	0.01

### Cell response

**FIGURE 2:**
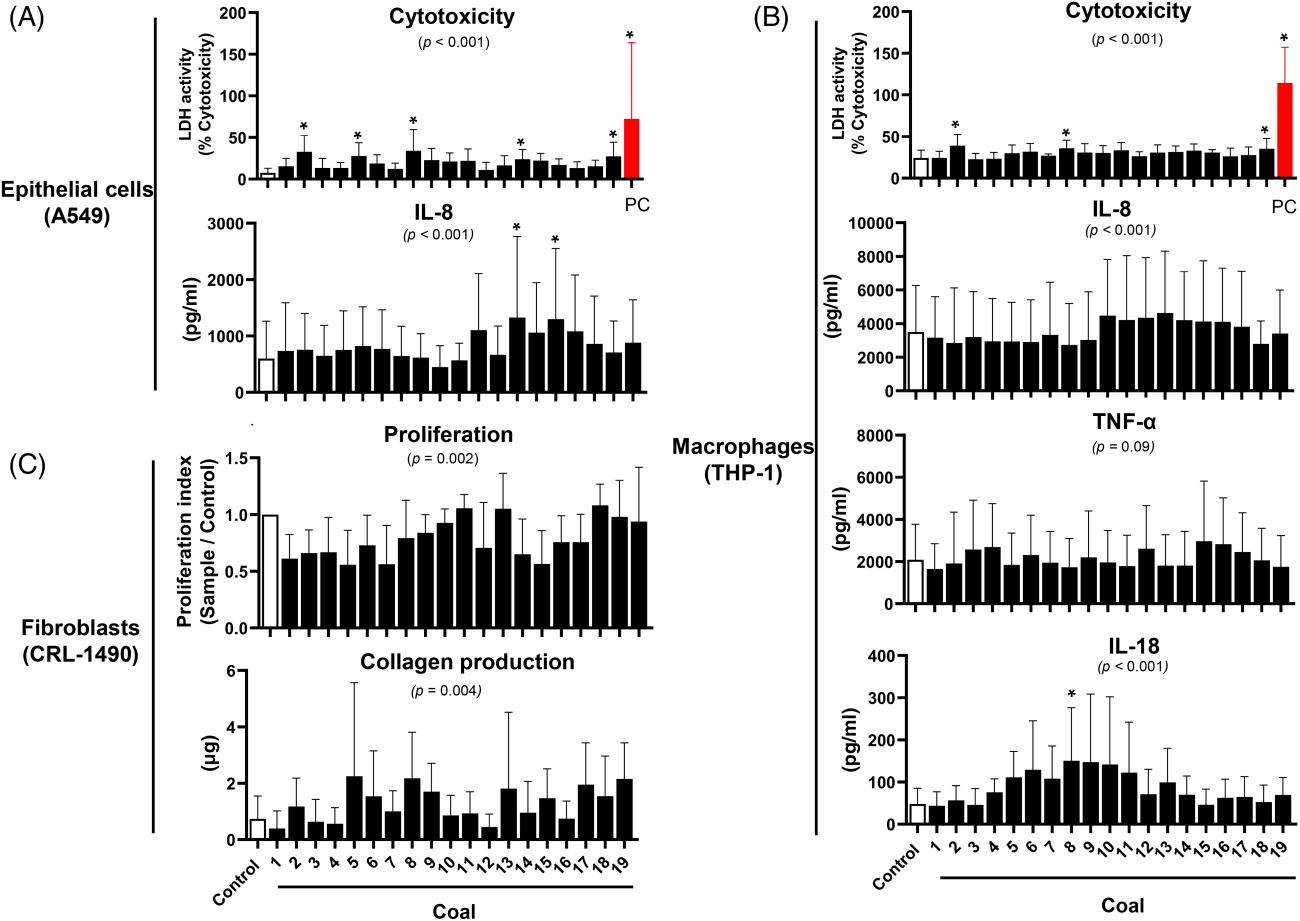
Cellular response. Cytotoxicity and cytokine production were assessed in in A549 (A) and THP‐1 cells (B), while proliferation and collagen production were quantified in CRL‐1490 cells (C) in response to 19 different coal samples. *p <0.05, compared to the control group. The overall ANOVA *p*‐values are shown in the graph. Values are mean (SD) with *n* = 6 per group for A549 and *n* = 7 per group for THP‐1 and CRL‐1490 cells. LDH, lactate dehydrogenase; PC, positive control for LDH assay

The cell response also varied between coal samples. We found (1) significant variation in cytotoxic activity (*p* <0.001) and IL‐8 secretion (*p* <0.001), with coals 2, 5, 8, 14 and 19 increasing cell death and coals 13 and 15 increasing IL‐8 production in A549 cells compared to control (*p* <0.05 for all comparisons) (Figure 2A); (2) significant variation in cytotoxic activity (*p* <0.001), with coals 2, 8 and 19 increasing cell death in THP‐1 cells compared to control; (3) significant variation in inflammatory cytokine production in THP‐1 cells including IL‐8 (*p* <0.001) and IL‐18 (*p* <0.001), but not TNF‐α (*p* = 0.09), although, post hoc analysis did not identify specific samples that were particularly detrimental with the exception of coal 8 for IL‐18 production (Figure 2B); and (4) significant variation in proliferation (*p* = 0.002) and collagen production (*p* = 0.004) in CRL‐1490 cells; post hoc analysis did not reveal any specific samples that were different to control (Figure 2C). IL‐6 levels in A549 and THP‐1 cells were below the detection limit of the assay.

As cytotoxicity and IL‐8 were performed in parallel on the same samples, we are able to analyse the associations between these two cellular events. Indeed, there was a significant linear relationship between cytotoxicity and IL‐8 production in A549 (*r* = 0.515, *p* <0.0001) and THP‐1 (*r* = 0.120, *p* = 0.004) cells.

### Associations between the cellular response and the coal characteristics

As the pathogenesis of CWP is likely to involve multiple cell types,^4^, ^5^, ^6^ we used PCA to group cellular responses that clustered together to study their association with coal chemistry. PCA generated three factors accounting for 34%, 23% and 18% of the variance, respectively. These factors were strongly loaded on cytotoxicity of A549 and THP‐1 cells, collagen production and THP‐1 TNF‐α production (PC1); IL‐8 production in A549 and THP‐1 cells (PC2); and CRL‐1490 proliferation and collagen production (PC3) (Table 2). Thus, PC1, PC2 and PC3 broadly represent cell cytotoxicity, inflammation and fibrotic processes, respectively.

**TABLE 2:** PCA loadings for the eight cellular outcomes assessed with the loadings for each outcome

Variables	PCA1 (34%)	PCA2 (23%)	PCA3 (18%)
THP‐1 cytotoxicity	0.881		
A549 cytotoxicity	0.844		
CRL‐1490 collagen	0.689		0.562
THP‐1 TNF‐α	−0.672		0.390
THP‐1 IL‐18	0.487		
A549 IL‐8		0.937	
THP‐1 IL‐8		0.933	
CRL‐1490 proliferation			0.940

Abbreviation: PCA, principal component analysis.

**FIGURE 3:**
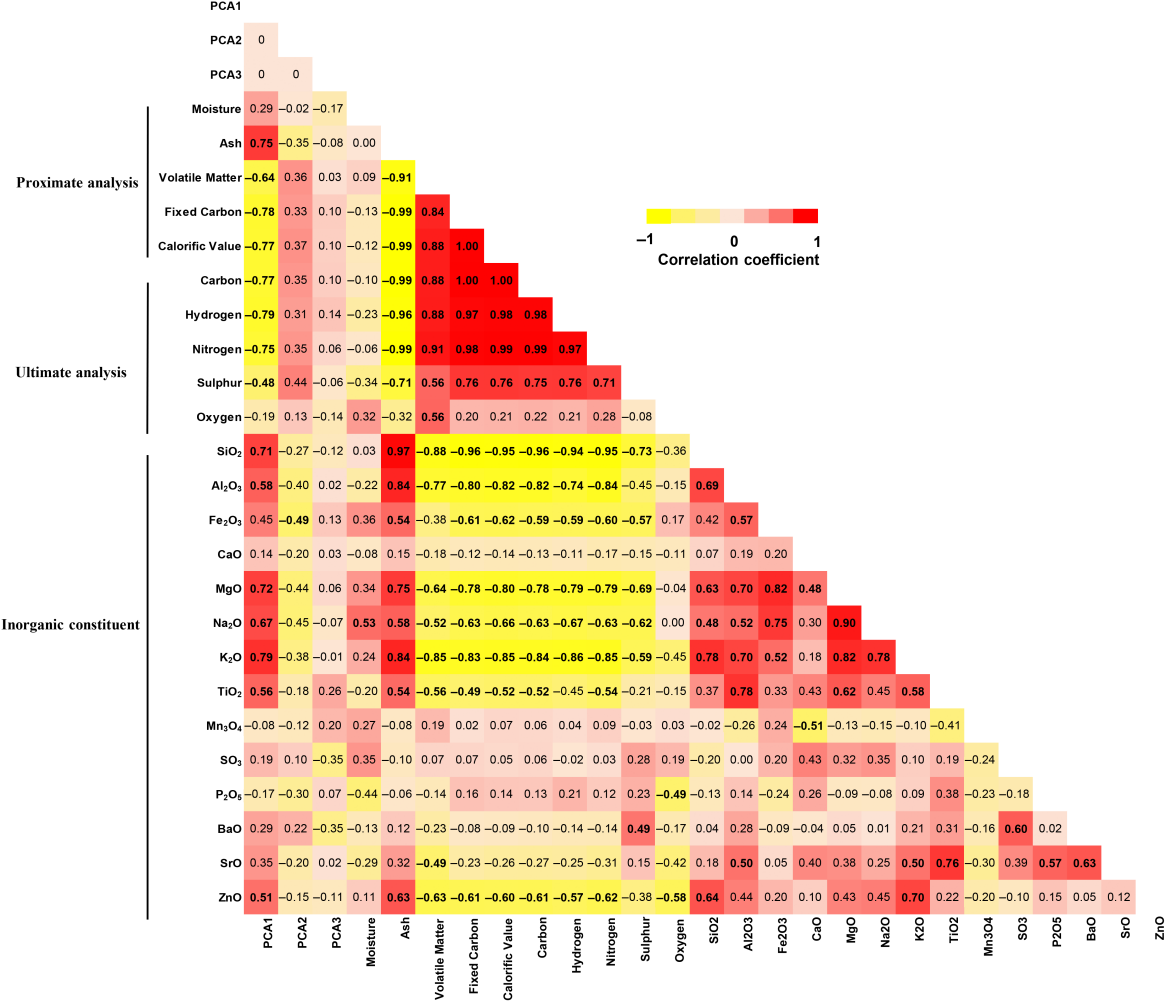
Correlation between particle constituents and cellular response. The correlation matrix shows the correlations between coal compositions and scores PC1, PC2 and PC3 which reflect cell cytotoxicity, the inflammatory response and fibrotic response, respectively. Each cell in the table shows the correlation coefficients between the two variables, with the corresponding colour intensity indicating the strength of the association. Numbers in bold indicate a significant correlation between the two variables (*p* <0.05). PCA, principal component analysis

PC1 was inversely correlated with the major components (fixed carbon and carbon) and elements (hydrogen, nitrogen and sulphur) of the coal and positively associated with ash content (*r* = 0.75, *p* <0.01) and a range of chemical constituents including SiO_2_, Al_2_O_3_ MgO, Na_2_O, K_2_O, TiO_2_ and ZnO (*r* = 0.51–0.79, *p* <0.05 for all correlations). The high number of associations between PC1 and coal chemistry is likely due to the high level of collinearity between the chemical constituents (Figure 3). In contrast, PC2 was only found to be negatively correlated with Fe_2_O_3_ (*r* = −0.49, *p* <0.05), while no significant association was identified for PC3.

To deal with the high level of collinearity, we applied stepwise multiple regression analysis to identify the most important coal components that were associated with the detrimental cellular effects. Using this approach, K_2_O explained 62.8% of the variance in PC1 (*p* <0.0001), while two variables (Fe_2_O_3_ and P_2_O_5_) were retained in the model accounting for 41.5% of the PC2 variance (*p* = 0.014) (Table 3). In line with the univariate analysis, stepwise multiple regression did not reveal any variables associated with PC3. The identified associations were largely unaltered after adjustment for particle size in the linear regression model, with the exception of P_2_O_5_ (*p* = 0.11) (Table 3).

**TABLE 3:** Significant associations between coal chemistry and the cell response identified using stepwise multiple regression. Multivariate linear regression was applied to determine whether these associations were maintained after adjustment for particle size

	Stepwise regression analysis				Linear regression analysis		
Cell response	R^2^ change	*β*	ANOVA (*F*)	*p*	*β*	95% CI	*p*
PC1							
K_2_O	0.628	0.792	28.678	<0.0001	0.821	0.489–1.154	<0.0001
PC2							
Fe_2_O_3_	0.235	−0.588	5.673	0.014	−0.427	−0.739 – −0.116	0.011
P_2_O_5_	0.180	−0.436			−4.622	−10.260 – 1.116	0.107
PC3	—	—	—	—	—	—	—

Abbreviation: PC, principal component.

## DISCUSSION

We sourced a range of real‐world coal samples with varying chemical compositions, in order to investigate the relationship between the chemical characteristics of coal particles and the cellular response in a range of cell types with direct relevance to the pathogenesis of CWP. We showed that the magnitude of the cytotoxic response and cytokine production in epithelial cells and macrophages, as well as the fibroblast response, varied considerably between coal samples. We were able to establish an association between the magnitude of these responses and the chemical composition of the coal particles. In particular, we identified a strong positive association between cytotoxicity and K_2_O, and a negative association between inflammation and Fe_2_O_3_. Thus, our data clearly demonstrated that the detrimental effect of coal particles on lung cells is associated with the chemical constituents of the particles.

Exposure to coal particles induced cell death and inflammation in epithelial cells and macrophages, as well as increased collagen expression in fibroblasts. All of these features are well known to contribute to the pathogenesis of CWP. Upon inhalation, coal particles directly interact with epithelial cells and resident macrophages leading to the release of lipases and proteases which activate apoptotic processes.^6^ Inflammatory cytokines, particularly those secreted by alveolar macrophages, exacerbate cellular damage, initiate tissue injury and promote fibrogenesis.^4^, ^5^ The cytokines assessed in the present study are associated with the pathogenesis of CWP and are biomarkers of disease severity.^4^, ^6^, ^19^ In addition to responding to the mediators released by primary target cells,^5^ fibroblasts may come into direct contact with coal particles via interstitial particle translocation.^6^ We were able to establish that coal particles have a direct effect on fibroblasts by inducing collagen production and decreasing cell proliferation. These cytotoxic effects on fibroblasts are likely to be counteracted by the potent growth effects of mediators released by resident macrophages.^5^, ^20^ Further studies are needed to determine the fibrogenic effects of these particles in the context of cell–cell interactions.

Importantly, our data showed that the intensity of the cell response varied considerably between coal samples. Given that particle size was relatively uniform, it is likely that this variability in the cellular response is primarily due to the underlying variations in the chemistry of the coal particles. This was confirmed by our regression approach which included adjustment for particle size. However, identifying the components of the coal that are most likely to be driving the detrimental cellular responses is challenging due to the high level of collinearity within the cellular responses, as highlighted by the positive association between cytotoxicity and IL‐8 production, and between the chemical compounds within the coal samples.

To address this issue, we combined PCA and stepwise regression to identify the chemical components of the coal that explained the bulk of the variance in the response. Using this approach, we found a strong association between the K_2_O content of the coal and cell cytotoxicity. Early studies suggest an aetiological role for aluminium‐potassium silicates in the pathogenesis of pneumoconiosis.^21^ The toxic effects of transition metals in biological systems have been well characterized.^22^ These elements have the ability to displace metals from their natural binding sites, interact with proteins and DNA, and produce free radicals and reactive oxygen species (ROS), resulting in apoptosis.^22^, ^23^ ROS can act as signalling molecules, which activate canonical inflammatory pathways including nuclear factor‐kappa B and AP‐1 and alter mitogenic and fibrogenic signals.^11^, ^24^ Thus, it is possible that these compounds induce detrimental cellular responses through regulation of ROS production. This is well aligned with the observations that the oxidative properties of coal mine dusts are primarily attributed to their transition metal constituents^11^ and ROS is increasingly recognized as the key signalling molecule in coal mine dust‐induced toxicity.^6^, ^11^, ^25^, ^26^ In contrast, the cytokine response was negatively associated with Fe_2_O_3_ and P_2_O_5_, although the latter was no longer significant after correcting for particle size. This is consistent with in vivo studies showing that increasing concentrations of Fe in the geogenic particles were inversely associated with the acute inflammatory response in mice.^27^ The negative response induced by Fe is intriguing, and it has been proposed previously that the pathogenesis of inorganic dust‐induced lung disease is due to the interaction of multiple compounds.^27^, ^28^ Our correlation data showed that the organic coal components were inversely associated with the inorganic compounds. Thus, it may be the ratio of these compounds that is the key driver of the cellular response, rather than an individual chemical component. Finally, we did not observe any direct associations between coal chemistry and the fibroblast response, implying that other constituents that we did not quantify (e.g., polycyclic aromatic hydrocarbons) may be responsible for this association.

Taken together, our data highlight the potential for K_2_O to be a marker of coal mine dust potency in terms of the risk they pose for CWP. While we did not conduct a mineralogical analysis of the samples to identify critical components of the coal deposit, these data highlight the need for further work in this area. Future work should focus on identifying the mineral components of coal deposits that are associated with these compounds and subsequent examination of links between these deposits and the prevalence of CWP. In addition, further work is required to confirm that the association is causal and identify the molecular processes driving the cellular response with a view to identifying potential therapeutic approaches. While the exposure protocol we used is well justified on the basis of previous work,^7^, ^16^, ^26^ a time course and dose–response study is needed to investigate whether the association between chemical characteristic of particle and cellular response remains consistent under a range of conditions. Nonetheless, our study used a systematic approach to identify associations between the chemistry of coal particles and the response in a range of lung cell types. Our data clearly highlight the work to be done in terms of understanding the pathogenesis of CWP and the role that coal chemistry plays in this process in order to reduce the burden of disease.

## CONFLICT OF INTEREST

None declared.

## AUTHOR CONTRIBUTION

**Katherine Southam:** Methodology (equal); validation (equal). **B. Basil Beamish:** Methodology (equal); resources (equal); writing – review and editing (equal). **Yong Song:** Data curation (equal); formal analysis (equal); writing – original draft (equal); writing – review and editing (equal). **Graeme R. Zosky:** Conceptualization (equal); formal analysis (equal); funding acquisition (equal); resources (equal); supervision (equal); writing – review and editing (equal).

## ETHICS APPROVAL DECLARATION

Not applicable.

## Supporting information

Figure S1‐Time course study of the cellular response

## Data Availability

The data that support the findings of this study are available from the corresponding author upon reasonable request.
