# Supplementary material for: Effects of chemical composition on the lung cell response to coal particles: Implications for coal workers' pneumoconiosis
Source: Respirology. 2022 Mar 20;27(6):447–54. doi: 10.1111/resp.14246 (PMC9314662; doi:10.1111/resp.14246)
Supplement: Supplementary file 1 — Figure S1‐Time course study of the cellular response [file RESP-27-447-s001.docx]

**Figure S1 – Time course study of the cellular response**


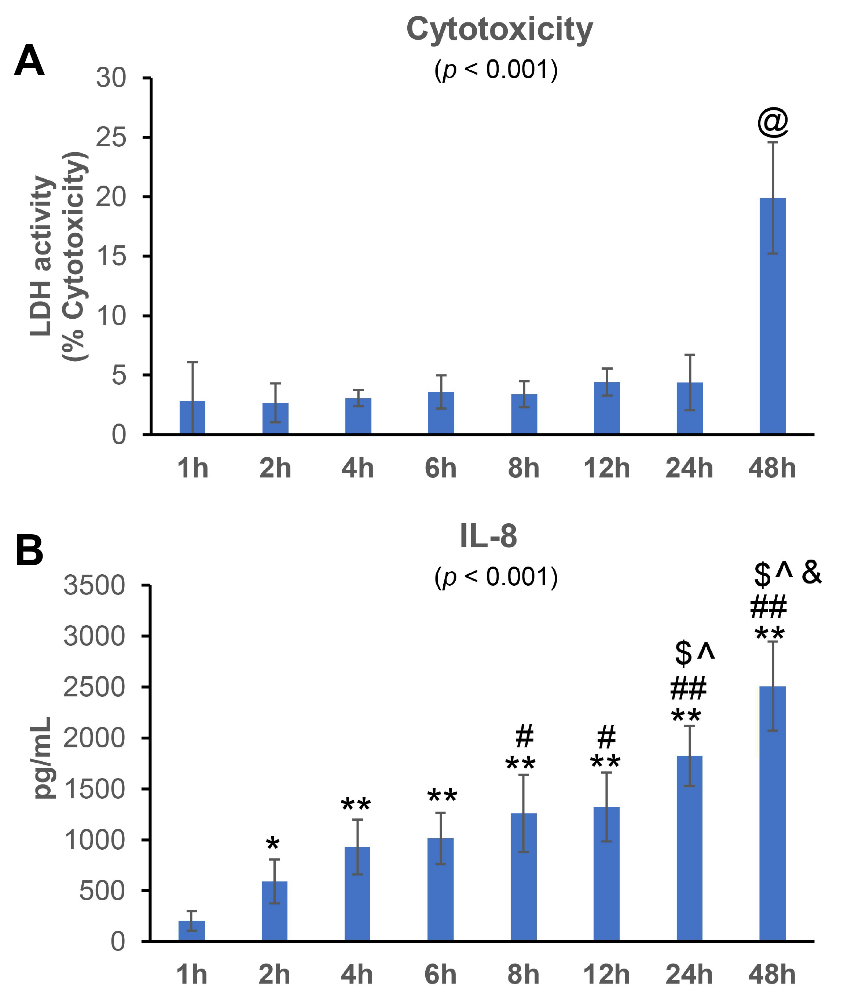


Cytotoxicity (A) and IL-8 production (B) were assessed in A549 in response to 200 µg/mL coal particles for 1 - 48 h. ^@^*p* < 0.001 compared to 1h – 24 h groups; ^*^*p* < 0.01, ^**^*p* < 0.001, compared to 1h group; ^#^*p* < 0.01, ^##^*p* < 0.001, compared to 2h group; ^$^*p* < 0.001 compared to 4h and 6h groups; ^^^*p* < 0.05 compared to 8h group; ^&^ *p* < 0.05 compared to 12h and 24h groups. The overall main effect ANOVA is shown in the graph. Values are mean (SD) with n = 6 per group.
